# Indoor Scene Point Cloud Registration Algorithm Based on RGB-D Camera Calibration

**DOI:** 10.3390/s17081874

**Published:** 2017-08-15

**Authors:** Chi-Yi Tsai, Chih-Hung Huang

**Affiliations:** Department of Electrical and Computer Engineering, TamKang University, 151 Yingzhuan Road, Tamsui District, New Taipei City 251, Taiwan; kuku6118@gmail.com

**Keywords:** point cloud registration, point cloud alignment, indoor scene reconstruction, multi-view calibration, RGB-D mapping

## Abstract

With the increasing popularity of RGB-depth (RGB-D) sensor, research on the use of RGB-D sensors to reconstruct three-dimensional (3D) indoor scenes has gained more and more attention. In this paper, an automatic point cloud registration algorithm is proposed to efficiently handle the task of 3D indoor scene reconstruction using pan-tilt platforms on a fixed position. The proposed algorithm aims to align multiple point clouds using extrinsic parameters of the RGB-D camera obtained from every preset pan-tilt control point. A computationally efficient global registration method is proposed based on transformation matrices formed by the offline calibrated extrinsic parameters. Then, a local registration method, which is an optional operation in the proposed algorithm, is employed to refine the preliminary alignment result. Experimental results validate the quality and computational efficiency of the proposed point cloud alignment algorithm by comparing it with two state-of-the-art methods.

## 1. Introduction

Three-dimensional (3D) scene reconstruction is an important issue for several applications of robotic vision such as map construction [1], environment recognition [2], augmented reality [3,4], and simultaneous localization and mapping (SLAM) [5,6]. Furthermore, 3D scene reconstruction usually requires numerous types of sensors such as stereo cameras, RGB-depth cameras, time-of-flight (TOF) cameras, lasers, and LIDAR. In this paper, we discuss how to employ RGB-D camera data for 3D scene reconstruction applications. RGB-D cameras use the structured light technique [7] to re-project the dispersed 3D data point set. The 3D colored point cloud information is obtained by using the RGB-D feature of combining two-dimensional (2D) RGB and depth images. Each frame of the RGB-D point cloud information has an independent coordinate system, which can be defined as the camera coordinate system *C_i_* at time *i*. When reconstructing a system, we expect to map each coordinate system *C_i_* to the same world coordinate system *W*, which serves as the same mapping target for each coordinate system *C_i_*. Therefore, in addition to defining a world coordinate system *W*, transformation matrices that transform each coordinate system *C_i_* to the world coordinate *W* are required. In our method, extrinsic parameters obtained from camera calibration are used to derive the transformation matrices which are then used to achieve a coarse reconstruction. Finally, some of the existing fine registration methods are employed to conduct the optimal 3D scene reconstruction.

Until now, several multi-view camera calibration studies have been performed with the aim of obtaining the transformation matrices for the transformation between different views. Transformation matrices have been used in numerous applications, for example, in a previous report [8], the bundle adjustment method was proposed to be employed to calibrate multi-view stereo cameras. In another report [9], the use of landmarks in a given scene as calibration references was proposed and the existing feature-matching methods were employed in the study to conduct multi-view landmark matching. Multi-view camera calibration was achieved in this manner, based on which a novel camera sequence method was suggested for integrating the information in an entire system and promoting the robustness of future applications. Li et al. [10] recommended using a feature description-based method, instead of the traditional checkerboard methods for camera calibration, by employing a special reference diagram as the camera calibration reference point in the feature description matching. In this study, we use the most common checkerboard multi-view camera calibration method to obtain the transformation relationships for different rotation angles, and apply them for point cloud registration. Holz et al. [11] documented the basic implementation method of using a point cloud library (PCL) to conduct registration. The method primarily involved capturing and aligning the feature descriptors to conduct an initial registration of the point clouds, and using the classic iterative closest point (ICP) [12] method to achieve fine registration. For all the operations, relevant application programming interfaces were provided by the PCL.

Local registration methods have proven to be able to generate an accurate reconstruction model if multiple point clouds are close enough to each other. Recently, several local registration methods with a higher accuracy have been proposed. Some studies [13,14] provide an overview of the fine and coarse registration methods. According to Reference [14], there are four types of local registration methods: the ICP method, Chen’s method [15,16], signed distance fields [17], and genetic algorithms [18]. Here, we chose the ICP method for our research because it is the most commonly used method in practice. The ICP method was proposed by Besl et al. [12] for the efficient registration of 3D shapes including free-form curves and surfaces. Subsequently, various extensions of the ICP method have emerged. Rusinkiewicz et al. [19] derived an ICP variant based on the uniform sampling of the space of normals which could provide comparatively good convergence results for relatively small scenes and sparse features. Low [20] suggested approximating the nonlinear least-squares optimization problem with a linear least-squares problem to solve the point-to-plane ICP algorithm. The author used an approximation method to increase the efficiency for determining the optimal solution. Serafin and Grisetti [21] formulated a new optimized cost function that included not only the point-to-point distance, but also surface normals or tangents; the new function was shown to not only increase the convergence speed, but also be advantageous in regard to the function robustness. For example, in a report [22] a system called KinectFusion was proposed; the system was based on a graphics processing unit (GPU)-accelerated ICP method and used a handheld moving Kinect to reconstruct indoor 3D scenes.

Feature descriptors are a technique that has been developed over a long time, evolving from the original 2D image feature descriptors to the current several 3D feature descriptors. Compared with 2D descriptors, 3D descriptors are richer in geometric information. Regardless of the computational efficiency decline owing to the increase in information, 3D descriptors can provide more accurate information regarding 3D objects or scenes. Rusu et al. [23,24] recommended the point feature histogram (PFH) descriptor, and further improved it to yield the fast point feature histogram (FPFH) [25] that was applied to point registration problems. FPFH significantly reduced the computational load of PFH by using some cache methods and modifying a few formulas; consequently, PFH was able to immediately achieve 3D point registration. Tombari et al. [26] recommended the signature of histograms of orientations (SHOT) descriptor, and reviewed some of the existing methods, classifying them as signatures and histograms methods. Then, the two types of methods were combined to generate a new 3D descriptor method, with the SHOT information lying between the signatures and histograms, and exhibiting both their features. Nascimento et al. [27] suggested a binary appearance and shape elements (BASE) descriptor that integrated both the intensity and shape information of a point cloud, and it was established in binary, thereby significantly enhancing the matching speed. Furthermore, the BASE descriptor could be applied to the scenes that were under poor lighting conditions and had sparse textures. Schmiedel et al. [28] proposed the interest-robust normal distribution transforms (NDT)-map (IRON) descriptor and applied it to robot localization; this descriptor was a feature descriptor used to reflect surface curvatures and object shapes, and it was established via NDT mapping.

On the other hand, a different way to reconstruct 3D indoor scenes using multiple RGB-D cameras can be interpreted as a fusion of depth maps obtained from different views, which is known as RGB-D image stitching [29,30]. In Reference [29], Song et al. proposed a rotated top-bottom dual-Kinect system to extend the field of view (FOV) of depth scanning based on the synchronized alignment of two RGB-D sensors. In other words, they employed two Kinects to maximize the FOV by stitching two depth images together to form a depth panorama; however, their system requires at least two RGB-D cameras to accomplish the RGB-D image stitching task. Recently, Li et al. [30] proposed an image-based RGB-D image stitching method that uses the registration data from color images to register depth maps. In other words, a calibration process is firstly conducted to find the relationship between the depth map and the color image, which is then used to align the depth map with the color image. By doing so, the problem of registering depth maps can be transformed into the problem of color image stitching, which usually requires significant computation and is difficult to run in real time.

From the above literature review, it can be seen that most of the modern point cloud alignment algorithms require a lot of computing power. With a greater amount of valid data in the point cloud, the required amount of computing will grow in a non-linear fashion. To improve the processing speed of point cloud alignment, this paper presents a novel calibration-based method which employs camera calibration techniques to find out the transformation matrices between some prefixed motor control points in the offline state, and then uses these transformation matrices directly in the online state. However, current camera calibration approaches usually induce calibration errors in the extrinsic transformation matrices, which may cause large errors in the registration result. To overcome this issue, an alignment calibration method is proposed to refine the calibrated transformation matrices. Unlike the FOV extension approaches in [29,30], the proposed method uses a single RGB-D sensor combined with a sequential panning-tilting capturing process to produce an accurate global registration result. Moreover, the computational load of the proposed method is very low because it only needs a simple coordinate transformation computation for each input point cloud data.

The following content describes the indoor scene reconstruction system that is proposed in this study. First, we introduce the related work in Section 2, including a simple description and classification of the current registration methods. Second, Section 3 systematically presents the processing steps of the proposed offline and online operations. Next, Section 4 provides the implementation details, including the offline mathematical models for system calibration and the coordinate system transformation methods for online operations. Last, we compare the details of several different point cloud alignment methods and present the results in Section 5. The conclusion is given in Section 6.

## 2. Related Work

Point registration has been a very actively researched topic. It is the process of performing a transformation to determine the mapping relationship between two point clouds in the 3D space. A transformation relationship can be expressed in terms of rotation and translation, using camera calibration models to describe how cameras project 3D scenes to 2D images. In the following expression for a camera calibration model

(1) $$s\left[\begin{array}{c} u\\ v\\ 1 \end{array}\right]=K\left[\begin{array}{cc} R_{w}^{c} & t_{w}^{c} \end{array}\right]\left[\begin{array}{c} x\\ y\\ z\\ 1 \end{array}\right]$$

we see that 2D-3D projection is determined by an extrinsic parameter matrix $\left[\begin{array}{cc} R_{w}^{c} & t_{w}^{c} \end{array}\right]\in {ℜ}^{3\times 4}$ and intrinsic parameter matrix **K**. Here, $R_{w}^{c}$ is a rotation matrix and $t_{w}^{c}$ is a translation vector from the world frame to the camera frame. Then, ${\left[\begin{array}{ccc} u & v & 1 \end{array}\right]}^{T}$ is a homogenous coordinate of a 2D pixel on the image plane, whereas ${\left[\begin{array}{cccc} x & y & z & 1 \end{array}\right]}^{T}$ is a homogenous coordinate of a 3D point in the world frame. s is a size ratio related to the depth of field, and it can be obtained from the depth images of a RGB-D camera.

Assume that there are two point data $p_{i}\in P$ and $q_{j}\in Q$, where $p_{i}={\left[x_{i},y_{i},z_{i}\right]}^{T}$ is the *i*th point of the point cloud **P** and $q_{j}={\left[x_{j},y_{j},z_{j}\right]}^{T}$ is the *j*th point of the point cloud **Q**. If there is a mapping relationship between **p***_i_* and **q***_j_*, the Euclidean distance can be expressed as $\left\|p_{i}-q_{j}\right\|$. To reduce the distance, we must define a cost function in terms of the rotation matrix $R_{p}^{q}$ and translation vector $t_{p}^{q}$ between two point clouds **P** and **Q**. According to Reference [31], there are three different types of point cloud registrations—namely global, local, and local descriptors registration—to provide the 3D coordinate rotation matrix $R_{p}^{q}$ and translation vector $t_{p}^{q}$. In the following section, we describe the difference between these three types of registrations and introduce their representative methods.

### 2.1. Local Registration

In this method, the distance between two point clouds should be short enough. Given a distance that is sufficiently short, a more accurate and rigid transformation may be obtained with the local registration of point clouds. Recently, the most dominant method has been the ICP method which determines the adjacent points in the two close point clouds P and Q, and defines a cost function $E_{p}^{q}$ as follows:

(2) $${E_{p}^{q}(R_{p}^{q},t_{p}^{q})|}_{p_{k}^{l}\in P,q_{k}^{l}\in Q}=\sum_{k=1}^{N}{\left\|(R_{p}^{q}p_{k}^{l}+t_{p}^{q})-q_{k}^{l}\right\|}^{2}$$

where $p_{k}^{l}$ and $q_{k}^{l}$ are the point correspondences that are sufficiently close to each other. *E_local_* is the sum of the errors of all the *N* point correspondences and *k* is the corresponding index. Then, an optimization method can be employed to determine the optimal *E_local_*. The advantage of this process is that it can evaluate the corresponding data points that are sufficiently close enough to each other, and thereby achieve a more accurate registration; however, this method has a prerequisite that the initial point clouds should be close enough to each other.

To minimize the cost function (Equation (2)), the random sample consensus (RANSAC) method can be implemented to iteratively obtain the optimal rotation matrix $R_{p}^{q}$ and translation vector $t_{p}^{q}$. This process is advantageous in that it is able to determine the corresponding point clouds, which are sufficiently close to each other and generate a registration that is more accurate. In addition to the requirement of a short enough distance between the two initial point clouds, this process also involves significant computation. Segal et al. [32] proposed a new plane-to-plane ICP method incorporating the original and point-to-plane ICP methods, and demonstrated that the new method could provide better experimental results than the previous two methods.

### 2.2. Global Registration

**Figure 1:**
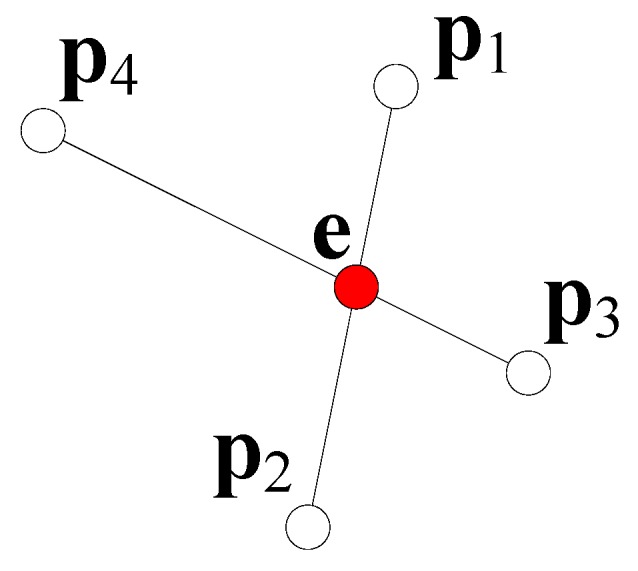
Schematic of the four-point algorithm in the four-point congruent sets (4PCS) method.

Global registration can be initiated with two point clouds in any status, without requiring them to be close enough to each other, and is able to obtain a result similar to local registration. Several methods belong to the category of methods globally searching the point correspondences between two sets of point clouds or image features. Alternatively, a few methods employ some geometric approaches to determine the local point correspondences, i.e., the four-point congruent sets (4PCS) method [33]. When the point correspondences in the point clouds **P** and **Q** are obtained, the cost function can be defined as ${E_{p}^{q}(R_{p}^{q},t_{p}^{q})|}_{p_{k}^{g}\in P,q_{k}^{g}\in Q}$, where $p_{k}^{g}$ and $q_{k}^{g}$ are the detected point correspondences. Next, the cost function $E_{p}^{q}$ can be obtained using an optimization method. The advantage of this process is that the point cloud registration can be conducted at a random position, but this process usually has a higher degree of computation complexity as compared with local registration. In Reference [33], the 4PCS method is proposed for global registration. Figure 1 illustrates a schematic of the four-point algorithm in the 4PCS method. This method randomly three points in a point cloud, referred to as **p**_1_, **p**_2_, and **p**_3_, and then uses a three-point plane to determine a fourth point **p**_4_. Subsequently, this method employs the cross-connected lines to obtain a cross point e, and calculates the ratio of the cross point distance $r_{1}=\left\|p_{1}-e\right\|/\left\|p_{1}-p_{2}\right\|$ and $r_{2}=\left\|p_{3}-e\right\|/\left\|p_{3}-p_{4}\right\|$. Given *r*_1_ and *r*_2_, all the points in the point cloud **Q** can now be connected, and the online candidates $e_{1}=q_{i}+r_{1}(q_{j}-q_{i})$ and $e_{2}=q_{i}+r_{2}(q_{j}-q_{i})$ can be calculated according to *r*_1_ and *r*_2_. When the candidates **e**_1_ and **e**_2_ overlap with each other, the four points that correspond to **P** can be evaluated. With the four points, it is possible to obtain the rotation matrix $R_{p}^{q}$ and translation vector $t_{p}^{q}$ in the 3D space. The advantage of global registration is that this method can conduct point cloud registration at a random position, but this method usually leads to larger registration errors as compared with local registration.

### 2.3. Local Descriptors Registration

Local descriptor registration requires a feature descriptors matching process to search for point correspondences in two point clouds, with the data of the two point clouds not required to be close to each other initially. By contrast, local and global registration methods search for point correspondences locally or globally under certain conditions without using feature descriptors, leading to an inefficient point correspondence determination process.

The feature descriptors usually contain information about point features in a local region such as surface, normal, signature, and texture. In this method, the feature descriptors may be extracted by different approaches to have the search capability for the point correspondences. By using feature descriptors to search for similar points that are the most close to one another in terms of the Euclidean distance, we can determine the point correspondences $p_{k}^{d}\in P$ and $q_{k}^{d}\in Q$ in two point clouds, with *k* as the corresponding index. Furthermore, we can use the point correspondences to define the cost function as ${E_{p}^{q}(R_{p}^{q},t_{p}^{q})|}_{p_{k}^{d}\in P,q_{k}^{d}\in Q}$.

A relatively classic descriptor is the PFH descriptor which extracts the translation invariance feature from a key point. This feature descriptor is a 3D data that is related to the angles between the key point and an adjacent point. In a study [26], further developments have been made to this feature descriptor to obtain the FPFH descriptor that was used in point registration problems. It was shown that the computation could be significantly reduced by using some cache methods and modifying formulas, and, therefore, the 3D point registration could be conducted instantly. Zhou et al. [34] recommended a fast global registration method based on FPFH which allowed rapid registration convergence; compared with some of the existing methods, this method was a more real-time computation while having a certain registration accuracy.

## 3. System Architecture

**Figure 2:**
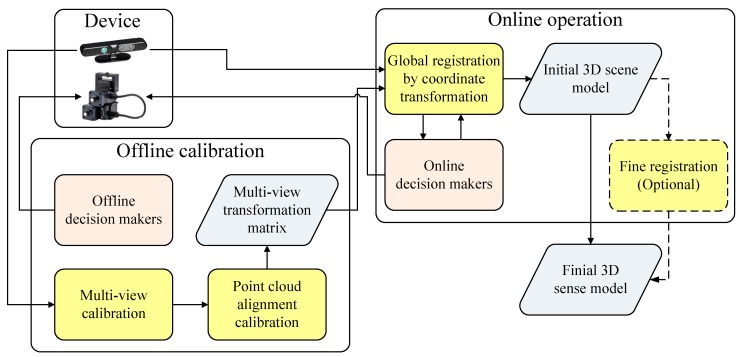
System architecture showing the 3D scene reconstruction method, consisting of three parts, namely device, offline calibration, and online operation.

**Figure 3:**
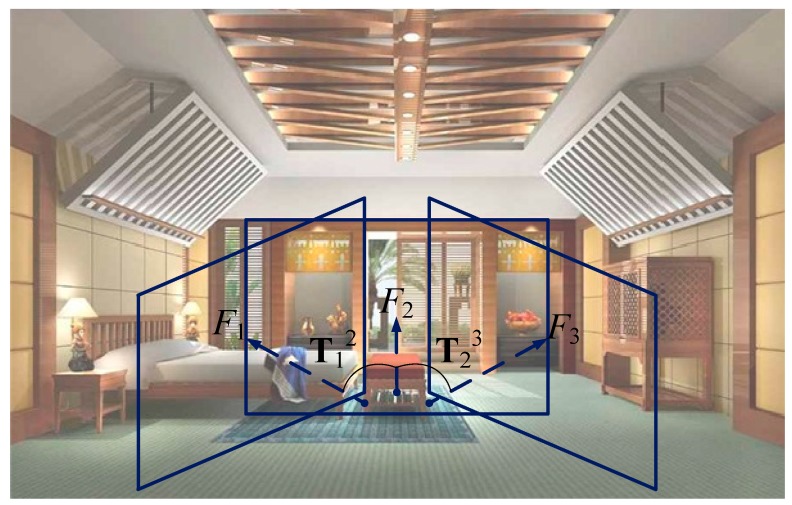
Schematic of the recording images obtained by rotating a device, with the blue rectangular areas representing the image planes. $T_{1}^{2}$ and $T_{2}^{3}$ are the two transformation matrices for the transformation between the two image planes.

In this section, we introduce the system architecture proposed in this study. Figure 2 illustrates the system architecture of the 3D scene reconstruction. First, the point cloud information for the scene reconstruction has to be obtained via device sensing. In an indoor scene, we can use a pan-tilt-driven RGB-D camera to record parts of the scene. It can be seen from Figure 3 that a camera driven by the pan-tilt records continuous frames *F*_1_, *F*_2_, and *F*_3_. Here, $T_{1}^{2}$ is the view transformation matrix to transform *F*_1_ to *F*_2_, whereas $T_{2}^{3}$ is the view transformation matrix to transform *F*_2_ to *F*_3_. *F_i_* contains the information of the RGB and depth images, which are transformed—with tools—to yield the color point cloud information **P***_i_* = {{**p**_1_, **c**_1_}, {**p**_2_, **c**_2_}, ...}. Here, **p***_j_* = [*x_j_*, *y_j_*, *z_j_*]*^T^* is the position of point *j* in a point cloud (in the *F_i_* coordinate) and **c***_j_* = [*r_j_*, *g_j_*, *b_j_*]*^T^* is the color of point *j* in the point cloud. Given that each *F_i_* has its own coordinate system, it is necessary to use the transformation matrix $T_{i}^{0}$ to combine those coordinates into one. Note that the proposed system does not include processes of feature matching, outlier removal, and motion estimation, because the required transformation matrices are obtained from the offline calibration. This design greatly reduces the processing time of 3D scene reconstruction and is also the main difference between the proposed method and the existing ICP-based methods.

### 3.1. Forward Kinematics Approach

**Figure 4:**
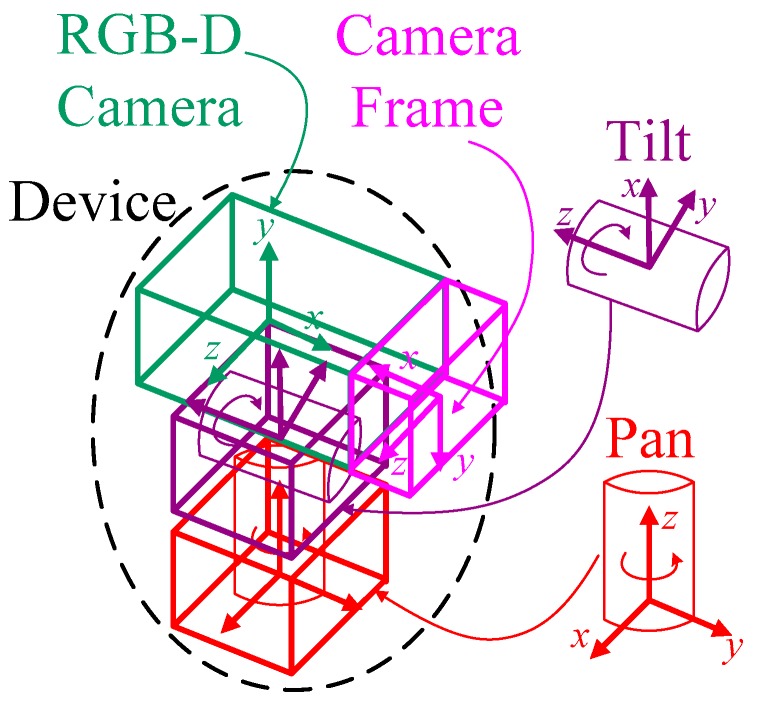
Motion model of the pan-tilt device used in the proposed 3D scene reconstruction system.

Because the RGB-D camera is driven by a pan-tilt device, the transformation matrix can be directly obtained from a forward kinematics analysis of the motion platform [35]. Figure 4 illustrates the motion model of a pan-tilt device considered in the proposed system. Because the coordinate transformation of the camera frame is determined by connection links of the pan-tilt unit, it is mandatory that the transformation between each connection should be taken into consideration. Given that the pan, tilt, RGB-D camera, and camera frames are all on different coordinate axes, the corresponding forward kinematics Equation can be derived from the conventional Denavit-Hartenberg (D-H) link parameter method [36], which defines four D-H link parameters: the link length *a_k_*, the link twist *α_k_*, the link offset *d_k_*, and the joint angle *θ_k_*. Table 1 shows the D-H link parameters of the pan-tilt device used in the experiment (see Section 5). According to Table 1, the transformation matrix between *k*−1 and *k* joint frames can be computed by

(3) $$\begin{array}{c} A_{k}^{k-1}=R_{z}(\theta_{k})T_{z}(d_{k})T_{x}(a_{k})R_{x}(\alpha_{k})\\ \;=\left[\begin{array}{cccc} c_{\theta_{k}} & -s_{\theta_{k}} & 0 & 0\\ s_{\theta_{k}} & c_{\theta_{k}} & 0 & 0\\ 0 & 0 & 1 & 0\\ 0 & 0 & 0 & 1 \end{array}\right]\left[\begin{array}{cccc} 1 & 0 & 0 & 0\\ 0 & 1 & 0 & 0\\ 0 & 0 & 1 & d_{k}\\ 0 & 0 & 0 & 1 \end{array}\right]\left[\begin{array}{cccc} 1 & 0 & 0 & a_{k}\\ 0 & 1 & 0 & 0\\ 0 & 0 & 1 & 0\\ 0 & 0 & 0 & 1 \end{array}\right]\left[\begin{array}{cccc} 1 & 0 & 0 & 0\\ 0 & c_{\alpha_{k}} & -s_{\alpha_{k}} & 0\\ 0 & s_{\alpha_{k}} & c_{\alpha_{k}} & 0\\ 0 & 0 & 0 & 1 \end{array}\right]\\ \;=\left[\begin{array}{cccc} c_{\theta_{k}} & -s_{\theta_{k}}c_{\alpha_{k}} & s_{\theta_{k}}s_{\alpha_{k}} & a_{k}c_{\theta_{k}}\\ s_{\theta_{k}} & c_{\theta_{k}}c_{\alpha_{k}} & -c_{\theta_{k}}s_{\alpha_{k}} & a_{k}s_{\theta_{k}}\\ 0 & s_{\alpha_{k}} & c_{\alpha_{k}} & d_{k}\\ 0 & 0 & 0 & 1 \end{array}\right] \end{array}$$

where $c_{\theta }\equiv \cos\theta$ and $s_{\theta }\equiv \sin\theta$. Based on the joint coordinate transformation in Equation (3), the forward kinematics Equation of the pan-tilt device can be obtained by chain multiplying four link transformation matrices such that

(4) $$T_{i}^{0}=\prod_{k=1}^{4}A_{k}^{k-1}=\left[\begin{array}{cccc} s_{\theta_{p}} & -c_{\theta_{p}}c_{\theta_{t}+90} & c_{\theta_{p}}s_{\theta_{t}+90} & a_{3}s_{\theta_{p}}+a_{2}c_{\theta_{p}}c_{\theta_{t}+90}\\ -c_{\theta_{p}} & -s_{\theta_{p}}c_{\theta_{t}+90} & s_{\theta_{p}}s_{\theta_{t}+90} & a_{2}s_{\theta_{p}}c_{\theta_{t}+90}-a_{3}c_{\theta_{p}}\\ 0 & -s_{\theta_{t}+90} & -c_{\theta_{t}+90} & d_{1}+a_{2}s_{\theta_{t}+90}\\ 0 & 0 & 0 & 1 \end{array}\right]$$

where *θ_p_* and *θ_t_* denote the pan angle and the tilt angle of the device, respectively. Therefore, the transformation matrix in Equation (4) can be used to align the point cloud at the frame *F_i_* to *F*_0_ directly.

### 3.2. Camera Calibration Approach

Another way to obtain the transformation matrix information of each *F_i_* coordinate frame pertains to the offline calibration of the device. In this approach, the motor must be rotated to generate different camera views, and the rotation control is achieved via offline decision-makers. The following are the steps for the offline calibration operations:

1. Start to capture the initial frame *F*_0_, and define its coordinate as a world coordinate *W*.
2. Conduct multi-view calibration to obtain the extrinsic parameter matrix for the initial world coordinate.
3. Control the motor and allow it to rotate to the next point.
4. Capture the frame *F_i_*, and conduct multi-view calibration to obtain the extrinsic parameter matrix.
5. Generate point cloud **P***_i_*, and conduct point cloud alignment calibration to obtain the transformation matrix.
6. If completed, store all the transformation matrices; otherwise, go back to Step 3 to make a decision.

Therefore, prior to a scene reconstruction, we require conducting offline calibration to obtain multi-view transformation matrices. The implementation of offline calibration will be discussed in detail in Section 3.

With respect to online operation, we first develop a model for the 3D scene reconstruction. Because this system consists of a motor-controlled and point cloud-registered part, the online decision-makers are entirely responsible for the allocation and processing. Subsequently, 3D scene reconstruction is conducted according to the following steps:

1. Start to capture the initial frame *F*_0_ and generate point cloud **P**_0_, and define the coordinate of the point cloud **P**_0_ as the world coordinate *W*.
2. Control the motor and allow it to rotate to the next point, wait until the motor rotates to the fixed point, and then haul back the motor to complete the command.
3. Capture the frame *F_i_* and generate a point cloud **P***_i_*.
4. Transform the point cloud **P***_i_* to **P***_i_*^0^ using the corresponding transformation matrix **T***_i_*^0^ estimated in the offline calibration.
5. If completed, generate the initial 3D scene reconstruction model; otherwise, go back to Step 3 to make a decision of aligning the next point cloud.
6. If the fine registration step is enabled, then use one of the existing ICP methods to refine the initial 3D scene reconstruction model; otherwise, use the initial 3D scene reconstruction model as the final result.
7. Generate the final 3D scene reconstruction model.

When the 3D scene reconstruction is complete, the outlier points can be removed using some of the existing outlier removal methods [37,38]. The detailed operations of the point cloud registration will be presented in Section 4.

## 4. The Proposed Method

This study primarily proposes a 3D scene construction method that integrates motor control and camera calibration. The camera calibration is conducted via a camera to first detect and identify a key point in the checkerboard. With offline calibration, a relative transformation matrix for the camera in a fixed position can be obtained and used for global registration. In the following content, we present the offline calibration and global registration methods proposed by us, and also introduce how to integrate them with local registration.

### 4.1. Offline Calibration

**Figure 5:**
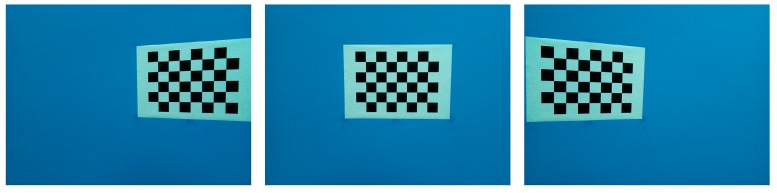
Illustration of recording images of a fixed reference diagram from different views. The images from left to right indicate the camera views that are rotated from left to right.

This subsection primarily introduces the proposed offline calibration method of generating multi-view transformation matrices based on multi-view camera calibration. Suppose that the intrinsic parameter matrix **K** can be obtained by fixing the extrinsic parameter matrix as $\left[\begin{array}{cc} I & 0_{3\times 1} \end{array}\right]$ (do not move the view) and using camera calibration [10]. Our method aims to obtain the extrinsic parameter matrix between the initial frame *F*_0_ and the *i*th frame *F_i_*. Figure 5 illustrates the different images in which the object (checkerboard) exists in each image captured from a different view. In this approach, the intrinsic parameters are fixed, whereas the extrinsic parameter matrices are obtained via camera calibration. Based on the extrinsic parameters, the following formula is obtained:

(5) $$\left[\begin{array}{c} P_{i}\\ 1 \end{array}\right]=\left[\begin{array}{cc} R_{r}^{i} & t_{r}^{i}\\ 0_{1\times 3} & 1 \end{array}\right]\left[\begin{array}{c} P_{r}\\ 1 \end{array}\right]=T_{r}^{i}\left[\begin{array}{c} P_{r}\\ 1 \end{array}\right]$$

where **P***_r_* is the coordinate of the key point on the checkerboard that is used as the coordinate system. $T_{r}^{i}$ is the transformation matrix to map **P***_r_* to **P***_i_*. With camera calibration, we can obtain the transformation matrices at different views. Next, we want to obtain the relative transformation matrix between two views. Provided that *F*_0_ is defined as the world coordinate *W*, we can assume that all **P***_i_* should be transformed to *F*_0_, and we thereby obtain the following formula:

(6) $${\left(R_{r}^{i}\right)}^{T}P_{i}-{\left(R_{r}^{i}\right)}^{T}t_{r}^{i}={\left(R_{r}^{0}\right)}^{T}P_{0}-{\left(R_{r}^{0}\right)}^{T}t_{r}^{0}$$

Then we expand and reorganize Equation (6) to form a complete transformation formula of **P***_i_* to *F*_0_ such that

(7) $$R_{i}^{0}P_{i}+t_{i}^{0}=R_{r}^{0}{\left(R_{r}^{i}\right)}^{T}P_{i}-R_{r}^{0}{\left(R_{r}^{i}\right)}^{T}t_{r}^{i}+t_{r}^{0}$$

where we define the rotation matrix $R_{i}^{0}=R_{r}^{0}{\left(R_{r}^{i}\right)}^{T}$ for the transformation of **P***_i_* to the *F*_0_ coordinate system and the translation vector $t_{i}^{0}=-R_{r}^{0}{\left(R_{r}^{i}\right)}^{T}t_{r}^{i}+t_{r}^{0}$. Finally, we calculate a transformation matrix of each view according to Equation (7), and thereby obtain all the transformation matrices as follows:

(8) $$T_{i}^{0}=\left[\begin{array}{cc} R_{i}^{0} & t_{i}^{0}\\ 0_{1\times 3} & 1 \end{array}\right]$$

**Figure 6:**
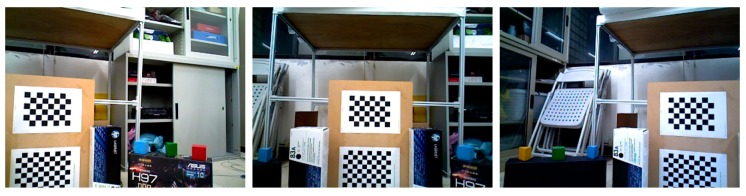
Illustration of the scene of point alignment calibration that is used in offline calibration. These scenes contain the same geometric objects.

These are the initial transformation matrices that are obtained by applying linear methods to the 2D point correspondences. However, these transformation matrices encounter calibration errors, which may deteriorate the registration result. To deal with this issue, we include fine registration in the offline calibration, a step that needs the identifiable geometric features of a scene. We can place some simple geometric objects in the scene as shown in Figure 6, in which each scene contains some geometric objects in addition to the board. The next step is to conduct 3D point alignment calibration, and this step requires local registration for conducting calibration. Here, we choose to use ICP. Because we can choose the way that the objects are placed in a scene to obtain good results, we do not need to select a complex method as long as the geometric objects are separated enough from one another and are representative. This part must be conducted using two adjacent point clouds. Suppose that point cloud $P_{i}^{0}$ which consists of each point **P***_i_* and **P**_0_ can be obtained after the transformation matrix in Equation (8), and, therefore, two adjacent point clouds can be denoted as $P_{i-1}^{0}$ and $P_{i}^{0}$. Next, with alignment calibration, we can obtain a refinement transformation between $P_{i-1}^{0}$ and $P_{i}^{0}$ as follows:

(9) $$P_{i-1}^{0}=R_{0}^{\left(i\right)}P_{i}^{0}+t_{0}^{\left(i\right)}$$

where $R_{0}^{\left(i\right)}$ and $t_{0}^{\left(i\right)}$ are the *i*th refinement rotation matrix aligning $P_{i}^{0}$ to $P_{i-1}^{0}$ in the *F*_0_ coordinate system and the *i*th refinement translation vector, respectively. Given that $P_{i}^{0}$ and $P_{i-1}^{0}$ are all in the *F*_0_ coordinate system, we can substitute Equation (7) into Equation (9) to form a new transformation relationship as follows:

(10) $$P_{i-1}^{0}=R_{0}^{\left(i\right)}R_{i}^{0}P_{i}+R_{0}^{\left(i\right)}t_{i}^{0}+t_{0}^{\left(i\right)}$$

According to Equation (10), we can obtain the aligned transformation matrix against the calibration errors such that

(11) $${\hat{T}}_{i}^{0}=\left[\begin{array}{cc} R_{0}^{\left(i\right)}R_{i}^{0} & R_{0}^{\left(i\right)}t_{i}^{0}+t_{0}^{\left(i\right)}\\ 0_{1\times 3} & 1 \end{array}\right]$$

Finally, the information of these transformation matrices is stored for use in the online operation.

### 4.2. Online Operation

This subsection primarily introduces how to use the multi-view transformation matrices to achieve initial and fine registration. Given that we have stored the transformation matrices in offline calibration, it will be easy for us to rapidly achieve initial registration. In Section 3, we introduced the initial world coordinate *W* and defined it as the coordinate of *F*_0_, while all the transformation matrices that we have obtained map to *F*_0_. First, we take *F*_1_ as an example. Provided that we want to let point cloud **P**_1_ of *F*_1_ be transformed to *F*_0_, we can use the following formula:

(12) $$P_{1}^{0}={\hat{T}}_{1}^{0}P_{1}$$

Here, $P_{1}^{0}$ is the transformed point cloud **P**_1_ in the *F*_0_ coordinate system. When the point clouds at all the views are processed by Equation (12), we can obtain an initial reconstructed 3D scene model $P_{w}=\{P_{0},P_{1}^{0},P_{2}^{0},\ldots ,P_{N}^{0}\}$, where *N* represents the number of multiple views.

Furthermore, an optional fine registration can be used to reduce the convergence error between the cloud point of **P**_0_ and that of **P**_1_. In other words, we conduct fine registration by using some existing local registration methods, such as ICP. After refining the transformation matrix, the new point cloud will be generated as shown below,

(13) $${\hat{P}}_{1}^{0}={\tilde{T}}_{0}^{\left(1\right)}P_{1}^{0}$$

where ${\hat{P}}_{1}^{0}$ is the point cloud **P**_1_ transformed to the *F*_0_ coordinate after local registration. ${\tilde{T}}_{0}^{\left(1\right)}$ is a refined transformation matrix that is generated with the fine registration of point cloud $P_{1}^{0}$ which has already been subjected to the initial registration. If the point clouds at all the views are treated according to Equations (12) and (13) and then combined into the same coordinate of *F*_0_, we can finally obtain a better reconstructed 3D scene model ${\hat{P}}_{w}=\{P_{0},{\hat{P}}_{1}^{0},{\hat{P}}_{2}^{0},\ldots ,{\hat{P}}_{N}^{0}\}$.

## 5. Experimental Results

**Figure 7:**
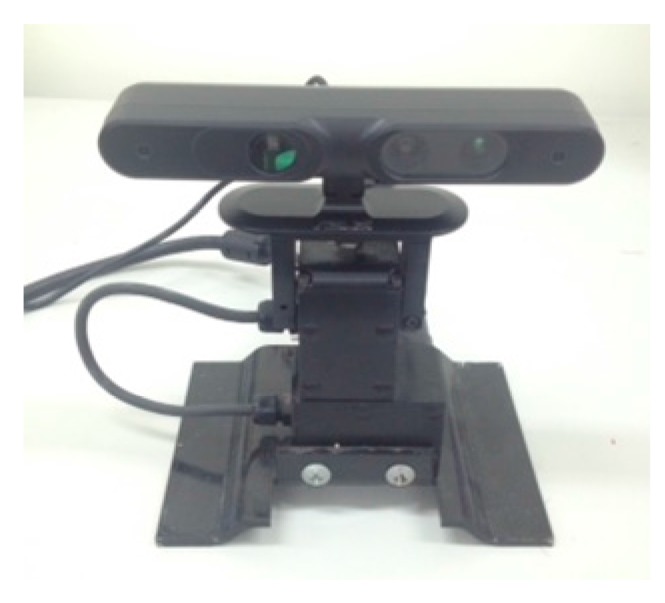
The physical pan-tilt device used in the experiments, which is an ASUS Xtion Pro RGB-D camera [39] mounted on an E46-17 pan-tilt platform [40].

Because the proposed algorithm is based on the combination of pan-tilt camera control and coordinate transformation to perform point cloud alignment, we did not use the existing public database in the experiment due to the requirement of offline calibration. A RGB-D camera and a pan-tilt platform were used in the experiment. We used the ASUS Xtion Pro RGB-D camera (ASUSTek Computer Inc., Taipei, Taiwan) [39] and mounted the RGB-D camera on the FLIR E46-17 pan-tilt platform (FLIR Motion Control System Inc., Goleta, CA, USA) [40], as shown in Figure 7. Table 2 shows the specifications of the FLIR E46-17 pan-tilt platform, which was used to drive the RGB-D camera for capturing indoor scene point clouds. Two state-of-the-art methods were employed in the experiment to compare with the proposed method. The first one is the fast global registration (FGR) method proposed in Reference [34], and the second one is the Super4PCS method proposed in Reference [31]. These two methods do not require offline calibration and usually can provide robust and accurate registration results. Moreover, we also employed the ICP algorithm as the refinement process for all methods used in the experiment. All experiments were carried out on an Ubuntu 14.04 notebook computer equipped with Intel Core i7-3530M CPU and 8 GB memory.

### 5.1. Point Cloud Registration Results

**Figure 8:**
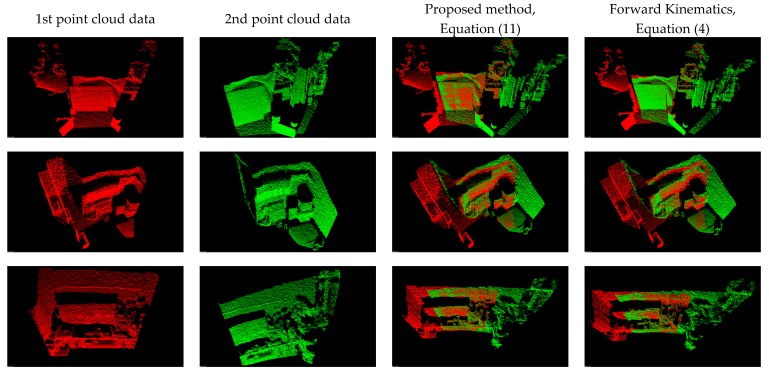
Comparison results between the proposed method and the forward kinematics method.

**Figure 9:**
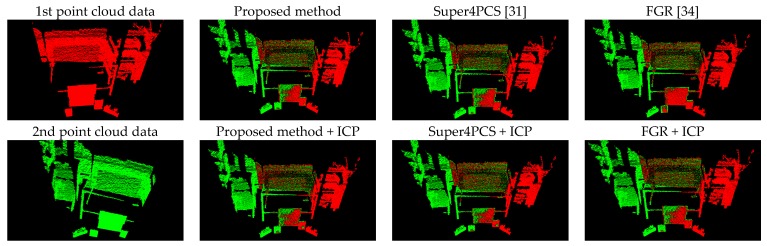
Experimental results of the 1 m dataset (a minimum of 1 m distance between the scene and the camera). The left column is the original point cloud data, the upper second column is the experimental result of the proposed method, and the lower second column is the result of the proposed method + ICP. The upper third column is the experimental result of Super4PCS, and the lower third column is the result of Super4PCS + ICP. The upper fourth column is the experimental result of FGR, and the lower fourth column is the result of FGR + ICP.

**Figure 10:**
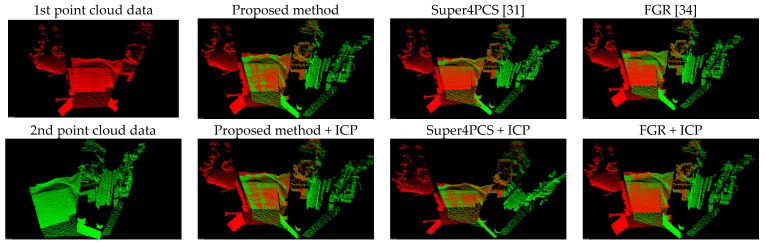
Experimental results of the Room 1 dataset.

**Figure 11:**
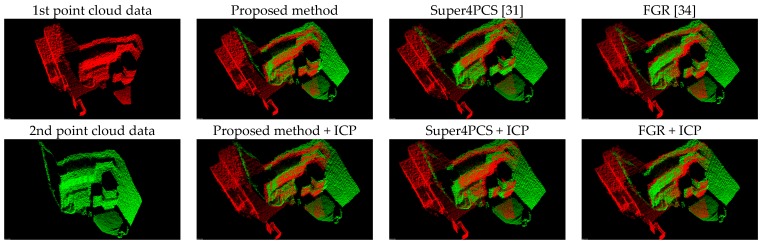
Experimental results of the Room 2 dataset.

**Figure 12:**
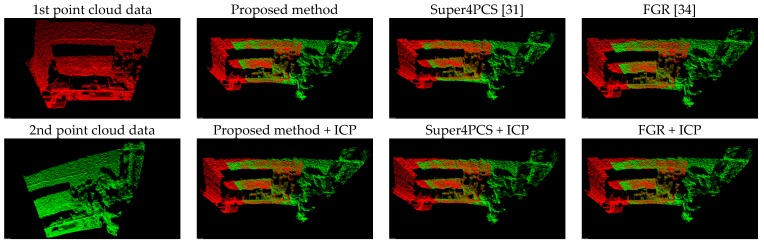
Experimental results of the Room 3 dataset.

**Figure 13:**
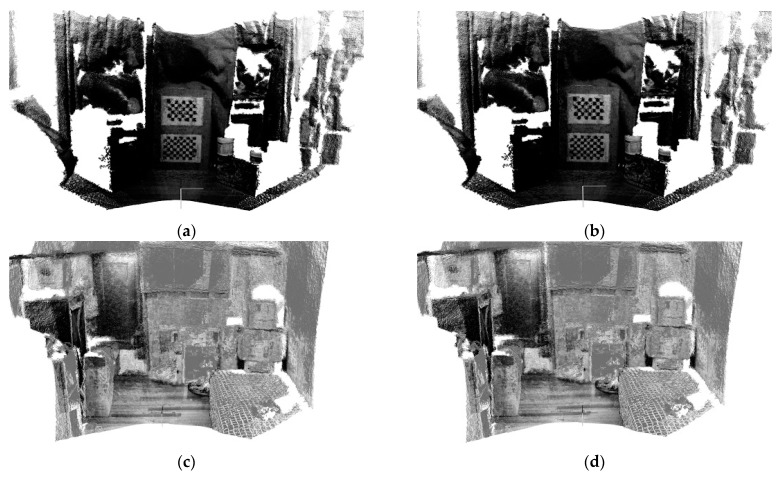

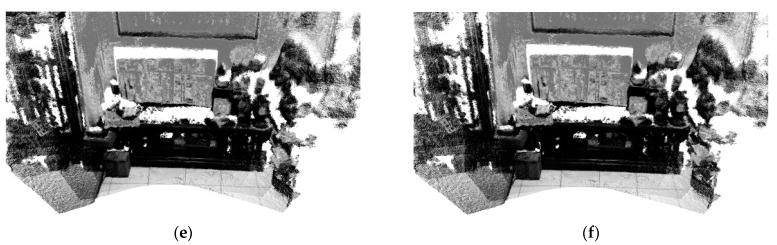
Registration results of the proposed method. (**a**) The initial registration result and (**b**) the refined registration result of the Room 1 dataset. (**c**) The initial registration result and (**d**) the refined registration result of the Room 2 dataset. (**e**) The initial registration result and (**f**) the refined registration result of the Room 3 dataset.

Figure 8 presents the comparison results between the proposed method (Equation (11)) and the forward kinematics method (Equation (4)). Visually observing Figure 8 finds that both methods provide similar point cloud registration results. Therefore, the correctness of the forward kinematics Equation (4) is verified. Figure 9 shows the experimental results of point cloud registration obtained from the proposed and the state-of-the-art methods. In this experiment, the minimum distance between the scene and the camera is about 1 m. In Figure 8, the pictures listed in the left column are two original point clouds captured from different viewers in the same scene. We can see from Figure 9 that all methods succeed in aligning the two point clouds. The pictures listed in the second column are the results of the proposed method, which illustrate that an initial registration result (the upper second column) can be obtained efficiently. The third column of the pictures presents the registration results of the Super4PCS method. Only using Super4PCS also can obtain an initial alignment result (the upper third column), which can be further coupled with the ICP method to make a refinement result (the lower third column). The right column of the pictures presents the registration results of the FGR method. The initial alignment result of the FGR method is shown in the upper right column, and it also can be improved by the ICP method to make the registration result better (the lower right column). Figure 10, Figure 11 and Figure 12 show the experimental results of three different rooms obtained from the proposed and the compared methods. One can see that the proposed method still provides a competitive point cloud registration performance in comparison to the Super4PCS and FGR methods. Figure 13 shows the registration results of the Room 1, Room 2, and Room 3 datasets obtained from the proposed method; each dataset contains 15 point clouds captured in different camera views that need to be aligned. One can see that the initial registration results of the proposed method are very close to the ICP refined ones. Therefore, the above experimental results validate the efficiency and robustness of the proposed method.

### 5.2. Quantitative Evaluation

To quantitatively evaluate the registration performance of the proposed and the compared methods, we employed the root mean square (RMS) metric [41] in the experiment as follows:

(14) $$RMS=\sqrt{\frac{\sum_{i=1}^{N}d_{\min}^{2}(i)}{N}}$$

where $d_{\min}\in {ℜ}^{N}$ is a vector containing the minimum distance (in millimeters) of *N* closest points between two aligned data scenes, and $d_{\min}(i)$ denotes the *i*th element of the vector $d_{\min}$. To evaluate the registration performance of the proposed aligned transformation matrix ${\hat{T}}_{i}^{0}$, Table 3 records three online registration comparison results between the Equations (4), (8) and (11) solutions. It is clear that the solutions of Equations (4) and (8) produce registration results with higher RMS errors due to modeling uncertainties and camera calibration errors, respectively. In contrast, the solution of Equation (11) produces better registration results against the calibration errors. Therefore, the registration performance of the proposed formula in Equation (11) is validated.

To investigate the effect of the minimum distance between the scene and the camera to the registration performance, we considered three cases, 1 m, 3 m and 5 m, in the experiment. Table 4 shows the average RMS errors of the registration results in these three cases. One can observe that the proposed method provides the best initial registration result for all three cases, followed by the FGR method and the Super4PCS method. After the fine registration process, the FGR method yields the best result, which is also very close to the refinement result of the proposed method. Furthermore, when the minimum distance between the scene and the camera increases from 1 m to 3 m, the RMS error of the proposed method just slightly increases about 0.2374 in average. In contrast, the average RMS errors of the two compared methods increase about 8.0790 and 0.4554 for the Super4PCS and the FGR methods, respectively. This implies that the proposed method is more robust to the variation of the distance between the scene and the camera when compared to the two state-of-the-art methods. However, when the minimum distance increases to 5 m, the average RMS errors of all methods increase rapidly above about 31. This is caused by the sensor limitation of the ASUS Xtion Pro RGB-D camera, whose effective sensing distance ranges from 0.8 m to 3.5 m. Therefore, in the case of the 5 m minimum distance, the captured point cloud has a large number of invalid data points, which significantly deteriorates the registration performance of the proposed and the compared methods.

Table 4 also records the RMS results of the three registration results shown in Figure 10, Figure 11 and Figure 12. It is clear from Table 4 that the proposed method produces the best initial registration results compared to the FGR method and Super4PCS method. On the other hand, the ICP refinement process only makes a minor improvement to the proposed method. Because the proposed method can provide accurate initial registration results, the ICP refinement process is an ignorable step in the proposed multi-view point cloud registration system.

### 5.3. Computational Efficiency

One major advantage of the proposed method is its high computational efficiency. Table 5 shows the average processing time of all the methods in the experiment. Observing Table 5 finds that the processing time of the proposed method is only about 4 ms regardless of the effect of distance and environment variations, which is much faster than the Super4PCS and the FGR methods to produce the initial registration result. However, the ICP refinement greatly increases the processing time of the proposed method. Therefore, without the ICP refinement process, the proposed method is able to accurately align multi-view point clouds in real time. Note that the average processing time of the proposed alignment calibration is about 52,914.98 ms. However, this step is only performed once in the offline calibration process.

## 6. Conclusions

In this paper, an efficient point cloud registration method is proposed to align multi-view point clouds based on a camera calibration technique. One advantage of the proposed method is that it converges quickly and only needs to perform point cloud transformations without any iterative process. The proposed method consists of an offline calibration and an online operation. In the offline calibration, the relationship between different fixed camera views is calibrated to form several specific coordinate transformation matrices. Moreover, an alignment calibration process is proposed to obtain more accurate transformation matrices against the calibration errors. In the online operation, the point clouds captured from these fixed camera views can be directly and accurately aligned through the corresponding transformation matrices. Experimental results show that the proposed method is able to produce accurate point cloud registration results, even if no fine registration process is performed. Furthermore, the proposed method can process in real time. The average processing time of the proposed method is only about 4 ms running on a commercial notebook computer to align two point clouds, which is much faster than the existing ICP-based point cloud registration approaches. These advantages can make the registration of multi-view point clouds possibly become an online process with high registration accuracy via a sequential panning-tilting capturing process of a single RGB-D camera.

In the future, the proposed method will be further extended to combine with different control platforms to facilitate a variety of 3D reconstruction applications, such as 3D dense-map building, object model reconstruction, etc.

## Figures and Tables

**Table 1:** D-H link parameters of the pan-tilt device used in the experiment.

k	α_k_ (deg)	a_k_ (mm)	d_k_ (mm)	θ_k_ (deg)
1	90	0	45	θ_p_
2	90	74.466	0	θ_t_ + 90
3	0	23	0	−90
4	0	0	0	180

**Table 2:** Specifications of the E46-17 pan-tilt platform [40].

Rated Payload	Maximum Speed	Position Resolution	Tilt Range	Pan Range
2.72 Kg	300°/s	0.013°	−47° to +31°	−159° to +159°

**Table 3:** Average RMS comparison results between forward kinematics, camera calibration, and the proposed methods.

Average RMS	Online Registration Methods		
Test Dataset	Forward Kinematics Equation (4)	Camera Calibration Equation (8)	Proposed Method Equation (11)
Room 1	18.7566	21.1770	18.4981
Room 2	26.6134	30.5964	26.3199
Room 3	19.5648	25.5299	18.3148

**Table 4:** Average RMS comparison results between the proposed and the state-of-the-art methods.

Test Dataset	Proposed Method	Super4PCS	FGR	Proposed Method + ICP	Super4PCS+ ICP	FGR + ICP
1 m dataset	19.6716	22.1680	19.7152	19.5366	20.3303	19.2841
3 m dataset	19.9090	30.2470	20.1706	19.6401	29.2774	19.0025
5 m dataset	32.0353	43.1703	33.9440	31.5003	40.6470	31.2734
Room 1	18.4981	27.0479	18.8040	18.3856	24.4929	18.4195
Room 2	26.3199	29.0571	29.5579	25.7527	25.6207	25.7157
Room 3	18.3148	20.9321	18.7524	18.2912	18.3036	18.2979

**Table 5:** Average processing time (in milliseconds).

Test Dataset	Proposed Method	Super4PCS	FGR	Proposed Method + ICP	Super4PCS + ICP	FGR + ICP
1 m dataset	4.11	553,958.70	12,077.14	27,378.52	599,331.00	70,038.29
3 m dataset	3.14	528,159.80	30,508.29	17,385.10	574,146.00	119,717.10
5 m dataset	4.18	100,250.80	48,501.62	32,572.52	237,221.20	65,188.71
Room 1	3.77	636,178.79	10,902.57	40,000.84	732,519.07	60,492.71
Room 2	4.24	135,305.92	57,163.71	52,331.24	261,104.14	160,310.5
Room 3	3.90	582,703.07	25,278.57	46,932.69	671,707.43	53,883.57
